# Inhibition of Zinc Dendrites Realized by a β-P(VDF-TrFE) Nanofiber Layer in Aqueous Zn-Ion Batteries

**DOI:** 10.3390/membranes12101014

**Published:** 2022-10-19

**Authors:** Geumyong Park, Hyeonghun Park, WooJun Seol, Seokho Suh, Ji Young Jo, Santosh Kumar, Hyeong-Jin Kim

**Affiliations:** 1 Graduate School of Energy Convergence, Institute of Integrated Technology, Gwangju Institute of Science and Technology (GIST), 123 Cheomdangwagi-ro, Buk-gu, Gwangju 61005, Korea; 2 School of Materials Science and Engineering, Gwangju Institute of Science and Technology (GIST), 123 Cheomdangwagi-ro, Buk-gu, Gwangju 61005, Korea; 3 Research Institute for Solar and Sustainable Energies (RISE), Gwangju Institute of Science and Technology (GIST), 123 Cheomdangwagi-ro, Buk-gu, Gwangju 61005, Korea

**Keywords:** artificial interface, coulombic efficiency, electrospinning, MnO_2_ cathode, PVDF

## Abstract

Uncontrollable Zn dendrite formations and parasitic side reactions on Zn electrodes induce poor cycling stability and safety issues, preventing the large-scale commercialization of Zn-ion batteries. Herein, to achieve uniform Zn deposition and suppress side reactions, an electrospun ferroelectric poly(vinylidene fluoride-*co*-trifluoroethylene) copolymer, a P(VDF-TrFE) nanofiber layer, is introduced as an artificial solid–electrolyte interface on a Cu substrate acting as a current collector. The aligned molecular structure of β-P(VDF-TrFE) can effectively suppress localized current density on the Cu surface, lead to uniform Zn deposition, and suppress side reactions by preventing direct contact between electrodes and aqueous electrolytes. The half-cell configuration formed by the newly fabricated electrode can achieve an average coulombic efficiency of 99.2% over 300 cycles without short-circuiting at a current density of 1 mA cm^−2^ and areal capacity of 1 mAh cm^−2^. Stable cycling stability is also maintained for 200 cycles at a current density of 0.5 A g^−1^ in a full-cell test using MnO_2_ as a cathode.

## 1. Introduction

In recent years, due to environmental pollution, climate change, and the energy crisis, the demand for environmentally friendly energy storage devices with high performance has dramatically increased [1,2,3,4]. Since 1991, when the first commercial lithium-ion batteries (LIBs) were revealed, LIBs have dominated the energy storage market and various industrial applications due to their longevity and high energy density [5,6,7]. However, due to the high cost, toxicity, and flammability of LIBs, environmentally friendly and nonflammable aqueous alternatives to LIBs have attracted considerable interest [8,9,10,11,12]. Among the various types of aqueous batteries, Zn-ion batteries (ZIBs) have been intensely studied as next-generation energy storage devices due to their high specific capacity (825 mAh g^−1^), low redox potential (−0.76 V vs. SHE), high abundancy of Zn metal, and low costs [13,14,15,16,17,18,19]. However, Zn dendrite formation during battery operation can cause an internal short-circuit and lead to severe problems in terms of cell performance and safety.

These dendrites are mainly formed by the accumulation of electric charges on the protrusion of the electrode to create a local current density, and the Zn ions are intensively electrodeposited at the same site, which is called the ‘tip effect’ [20]. In addition, parasitic side reactions such as the hydrogen evolution reaction (HER) and zinc hydroxide sulfate (ZHS) byproduct formation between the electrolyte and the electrode interface also adversely affect cell performance and safety [18]. Recently, the most popular strategy for mitigating localized current density and side reactions on Zn electrodes has been to introduce an artificial interface between the electrode surface and the electrolyte [19,21,22,23,24]. From the material aspect, polymers are suitable as artificial interface layers because they induce uniform Zn deposition through functional groups and offer better adhesion properties due to flexibility, which is advantageous in terms of electrode durability [25].

Poly(vinylidene fluoride) (PVDF) and its copolymer are known for their high dielectric constant (ε_r_ ≈ 10 at 1 kHz), superior mechanical strength, thermal stability, and chemical resistance [26,27]. In particular, highly polar β-crystalline phase PVDF-based polymers with all-trans conformations have outstanding ferroelectric properties among their various phases [28,29]. As a result of the aligned structure, β-PVDF has the high dipole moments between the hydrogen and fluorine moieties, which are aligned almost perpendicular to the carbon axis, and when an external electric field above a certain intensity is applied, electric displacement occurs [30,31,32]. Ascribed to these unique characteristics of β-PVDF-based polymers, many recent articles have focused on their potential application in energy storage devices [33,34,35,36,37]. There have been several attempts to apply the characteristics of β-PVDF in LIBs and ZIBs. Song et al. demonstrated that β-PVDF could induce high-rate Li-ion diffusion [33]. Hwang et al. reported that β-PVDF effectively drives dense and uniform Li deposition [34]. Wang et al. reported that modifying a typical Zn anode with a PVDF-based polymer can homogenize the current distribution and suppress parasitic side reactions on the Zn anode surface [38]. The critical point of these studies is that the intense polarization of β-PVDF on an electrode is advantageous for metal ion diffusion during battery cycling. However, since the thermodynamically stable α-phase is the dominant phase in raw PVDF materials, applying heat or an electric field is needed to form the β-phase from the α-phase [30,31,32].

Herein, we apply a poly(vinylidene fluoride-co-trifluoroethylene) copolymer, a [P(VDF-TrFE)] nanofiber layer (PNF), to achieve a high β-crystalline fraction. The introduction of trifluoroethylene (TrFE) to copolymerize with PVDF can increase the fraction of the β-phase compared to pure PVDF, ascribed to the strong steric hindrance of fluorine atoms [39]. Additionally, electrospinning using a high applied voltage is an efficient technique for forming a high β-crystalline fraction and is able to provide a highly porous nanofiber layer, which enables facile electrolyte penetration into the layer [29,40,41,42,43]. In our cell design, a uniform PNF layer is electrospun onto a Cu substrate acting as a current collector, which is known as a metal that has a high binding energy to Zn^2+^ ions [44]. As a result, during cell operation, the PNF layer acts as a passivation layer, and the oriented dipole moments vectors of the PNF along the localized electric field on the electrode surface effectively relieve the localized current density, thereby leading to uniform Zn deposition and achieving a superior lifecycle under various current densities.

## 2. Experimental Section

### 2.1. P(VDF-TrFE) and PVDF Nanofiber Fabrication

The electrospinning solution containing 11 wt.% P(VDF-TrFE) (FC30, Piezotech, Indianapolis, IN, USA) with MW = 450,000 g mol^−1^ (70:30 mol %) was prepared by dissolving the polymer in a 60:40 weight ratio of N,N-dimethylformamide (DMF) and acetone under intense magnetic stirring for 3 h at 50 °C. In addition, 1 wt.% pyridine formate buffer was added to the solution to increase the solution conductivity and to produce uniform and beadless nanofiber mats with an average fiber diameter of ~400 nm on copper foil wrapped over the drum collector. The thickness of the electrospun nanofiber layer was ~5 μm. The as-prepared solution was fed into a 12 mL plastic syringe attached to a 23-gauge steel needle and pumped to a spinneret using a syringe pump with a flow rate of 0.5 mL/h at 18 kV with a tip-to-collector distance (TCD) of 12 cm at 100 rpm. The as-spun nanofiber mat was annealed for 2 h at 120 °C in a vacuum oven before use to enhance the high β-crystalline phase. To prepare the PVDF nanofiber layer, a similar operating procedure was followed, except that 12 wt.% PVDF (Sigma Aldrich, St. Louis, MI, USA) with MW = 534,000 g mol^−1^ was employed at an applied voltage of 23 kV, with a flow rate of 0.8 mL/h and a TCD of 17 cm.

### 2.2. Preparation of MnO_2_ Electrode

The cathode slurry was prepared by mixing 70 wt.% MnO_2_ (US Research Nanomaterial, Houston, TX, USA), 20 wt.% Super C (MTI Co., Tokyo, Japan), and 10 wt.% PVDF (Solef 5130, Solvay, Brussels, Belgium). The slurry was cast on 30 μm Ti foil by the doctor blade method with a loading density of 1.9–2.1 mg cm^−2^.

### 2.3. Cell Assembly

The half cells were assembled using the Zn electrode and Zn@Cu or Zn@PNF-Cu electrodes. Two M ZnSO_4_ (Ducksan, Gwangju, Korea) in deionized water was employed as the electrolyte, and glass fiber filter paper (Watman, Saukkola, Finland) was employed as the separator. The symmetric cells were assembled using two identical Zn@Cu or Zn@PNF-Cu electrodes with the same electrolyte. Zn (5 mAh cm^−2^) was first deposited on Cu and PNF-Cu and cycled under various current densities with an areal capacity of 1 mAh cm^−2^. The full cells were assembled using a MnO_2_ cathode and Zn@Cu or Zn@PNF-Cu electrodes. Then, 2 M ZnSO_4_ + 0.1 M MnSO_4_ (Daejung Chemicals & Metals Co., Siheung, Korea) in DI water was used as the electrolyte to reduce the Mn^2+^ dissolution of MnO_2_. All the cells were assembled in an open atmosphere and kept at 25 °C for 24 h to allow electrolyte penetration after assembly.

### 2.4. Electrochemical Measurements

All electrochemical measurements were conducted using a two-electrode system assembled in CR2032-type coin cells (U&S Energy Co., Cheonan, Korea). A galvanostatic battery cycler (WBCS 3000, Wonatech, Seoul, Korea) was used to measure the cycling performance. The Zn plating/stripping process was conducted using a half cell. In the half-cell test, Zn was deposited onto the Cu electrode at 1 mA cm^−2^ for an hour and stripped to 1 V at the same current density, repeatedly. For long-cycle galvanostatic charge/discharge tests of symmetric cells and full cells, 5 mAh cm^−2^ Zn was deposited onto Cu and PNF-Cu electrodes at 1 mA cm^−2^ before cell assembly to provide a Zn source. Symmetric cells were charged/discharged under various current densities (0.1, 1, 5, and 10 mA cm^−2^) with an areal capacity of 1 mA cm^−2^. The electrochemical performance of the full cells was evaluated in a voltage range between 0.8 and 1.9 V at 0.5 A g^−1^. The LSV and CV measurements were carried out on a VSP-300 instrument (Biologic, Orlando, FL, USA). LSV for the Tafel plot was conducted at a scan rate of 0.5 mV s^−1^ with a voltage range from −0.1 to 0.1 V. CV for the half-cell test was conducted at a scan rate of 1 mV s^−1^ with a voltage range from −0.2 to 0.45 V. CV for the full-cell test was conducted at a scan rate of 0.1 mV s^−1^ with a voltage range from 0.6 to 1.8 V. Electrochemical impedance spectroscopy (EIS) measurements were conducted using a VSP-300 with an impedance frequency range of 100 kHz to 0.01 Hz and an alternating perturbation of ±10 mV. All electrochemical measurements were carried out in a 25 °C thermostatic chamber.

### 2.5. Material Characterization

The cycled cells were carefully disassembled for post-cycling measurements. The electrodes were rinsed using deionized water to remove residual electrolytes, followed by drying using pressed air to prevent oxidation. After disassembly of the cells, the PNF layer was carefully removed to observe the morphology of the deposited Zn. The morphologies of the materials were observed using FE-SEM (JSM-7500F, JEOL) and EDS (Ultim Max 65, Oxford Instrument, Abingdon, UK). XRD (D8 Advance, Bruker, Billerica, MA, USA) with a Cu Kα radiation source and FTIR (Vertex 70, Bruker, Billerica, MA, USA) were conducted to analyze the β-phase formation of the PNF layer. Contact angle measurement was executed to analyze the hydrophilicity of the electrodes using DI water at room temperature (25 °C). Electric field (P-E) hysteresis loops of the fabricated Pt/P(VDF-TrFE)/Cu capacitor were measured using a ferroelectric tester (Precision LC, Radiant Technology, Albuquerque, NM, USA) with triangular pulses with an amplitude of 180 V and a frequency of 2.5 kHz.

## 3. Results and Discussion

### 3.1. Characterization of the PNF-Cu Electrode

**Figure 1:**
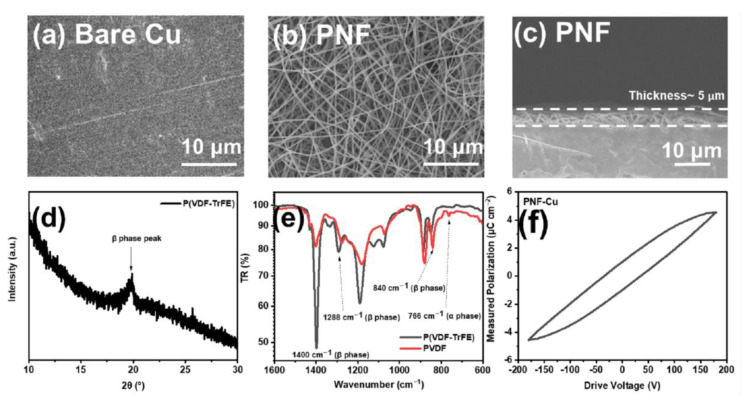
(**a**) Top-view SEM images of bare Cu foil and (**b**) PNF, (**c**) cross-sectional SEM image, (**d**) XRD pattern, (**e**) FTIR spectra, and (**f**) hysteresis loop of PNF.

Cu foil was selected as the current collector for P(VDF-TrFE) electrospinning (PNF-Cu). Figure S1 shows the nucleation overpotentials of the Zn‖Zn cell and the Zn‖Cu cell. When Zn ions are deposited on the Cu foil, they show a lower nucleation potential than when they are deposited on the Zn foil. This is due to the high binding energy between Zn^2+^ and Cu foil, suggesting that adopting a zincophilic Cu substrate can be an effective method to suppress Zn dendrite formation [34,45]. Figure 1a shows the surface of bare Cu foil: grooves or protrusions were observed on the surface, which led to the formation of dendrites by creating an environment where Zn ions can be intensively electrodeposited [46]. 

An electrospun PNF layer was introduced on the Cu foil surface to suppress dendrite formation, and Figure 1b,c show the morphology and thickness of the PNF layer, respectively. PNFs with a diameter of ~400 nm and a layer thickness of ~5 μm were uniformly electrospun on the Cu substrate. To verify that the PNF layer has a crystalline β-phase for ferroelectricity, the β-phase crystallinity was determined using X-ray diffraction (XRD). Figure 1d shows the characteristic peak of β-P(VDF-TrFE), which is located at ~20°, revealing that the crystalline β-phase of P(VDF-TrFE) was effectively formed [47]. To induce higher crystalline β-phase, heat treatment of the PNF layer was conducted at 120 °C for 2 h after the electrospinning process [45]. Equation (1) was used to calculate the β-phase content in the PNF layer through the FTIR spectra (Figure 1e) [48]:

(1) $$F\left(\beta \right)=\frac{A_{\beta }}{\left(\frac{K_{\beta }}{K_{\alpha }}\right)A_{\alpha }+A_{\beta }}$$

where *F(β)* is β-phase content, *K_α_* and *K_β_* are the absorption coefficients for each phase, which were 7.7 × 10^4^ and 6.1 × 10^4^ cm mol^−1^, respectively, and *A_α_* and *A_β_* are the absorbance peaks of nonpolar α and polar β phases at 766 and 840 cm^−1^, respectively.

The β-phase ratio of PNF was calculated to be 94%, which is generally higher than that when heat-treated in the form of a film, indicating that the β-phase was effectively generated through electrospinning [49,50]. On the other hand, the β-phase ratio of the electrospun PVDF nanofiber layer under a similar fabrication process was 83%, which shows the ease of β-phase formation of PNF due to the introduction of TrFE (Figure 1e). The polarization vs. electric field (P-E) hysteresis loop in Figure 1f shows that the dielectric response of the PNF layer under an external electric field can limit the localized current density on the tip of the protrusion, which is a key factor in reducing the dendrite growth. When the external electric field was removed, very low remanent polarization was observed (≈1.0 μC cm^−2^), which infers that it had a limited effect on increasing the overpotential during the cell operation.

**Figure 2:**
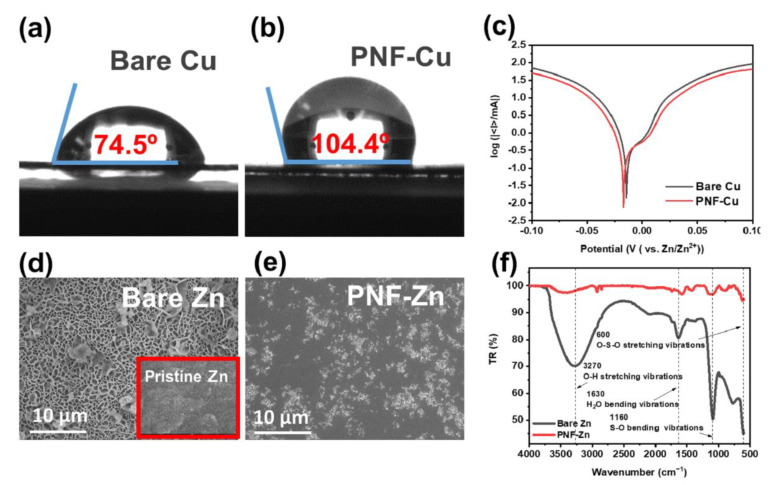
Contact angle measurement for aqueous solutions on (**a**) bare Cu and (**b**) PNF-Cu, (**c**) linear polarization curves showing the corrosion on bare Cu and PNF-Cu, and top-view SEM images of (**d**) bare Zn after immersion in the electrolyte for 7 days and pristine Zn (inset), (**e**) PNF-Zn after immersion in the electrolyte for 7 days, and (**f**) FTIR spectra of the bare Zn and PNF-Zn after immersion in the electrolyte for 7 days.

To check the compatibility with the electrolyte, contact angle and EIS measurements were conducted. The bare Cu substrate and PNF-Cu contact angles are 74.5° and 104.4°, respectively, as determined using deionized water (DI water) at room temperature (Figure 2a,b). The high contact angle of the PNF-Cu electrode is due to the hydrophobic properties of the PNF layer. The ionic conductivity of the PNF layer was measured by EIS measurements with a Cu symmetric cell and PNF-Cu symmetric cell (Figure S2). Despite the hydrophobic property of PNF, it exhibited an ionic conductivity of 2.59 × 10^−2^ S cm^−1^, which is large enough for Zn^2+^ conduction, which was achieved due to the small thickness and porous structure of the PNF layer [19]. Since very thin PNF was introduced on the Cu foil, there was a very slight increase in the bulk resistance (R_b_). The steeper slope in the low-frequency region of the PNF-Cu electrode than that of the bare-Cu electrode indicates the capacitive response from the high dielectric constant of the P(VDF-TrFE), and the polarity of the PNF layer possibly alleviates the localized current density around the protrusion of the electrode.

### 3.2. Suppression of Side Reactions

The hydrophobic property of the PNF layer can effectively block direct contact between the electrode and the electrolyte, which is expected to suppress the HER and the generation of byproducts. Linear sweep voltammetry (LSV) was conducted to evaluate the HER reaction behavior of the PNF-Cu electrode. The Tafel curves measured in 2 M ZnSO_4_ at a scan rate of 0.5 mV s^−1^ showed a more negative corrosion potential and lower current of PNF-Zn (Figure 2c) than those of the bare Cu electrode, suggesting that the PNF layer can effectively suppress the HER.

**Figure 3:**
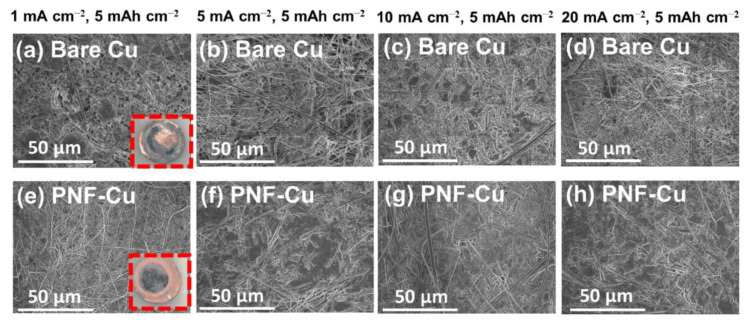
Top-view SEM images of Zn deposition morphology on (**a**–**d**) bare Cu electrode and (**e**–**h**) PNF-Cu under various current densities and fixed areal capacity of 5 mAh cm^−2^.

Additionally, to investigate the corrosion resistance property of PNF in the electrolyte, bare Zn foil and Zn foil electrospun with PNF (PNF-Zn) were immersed in 2 M ZnSO_4_ electrolyte for 7 days, followed by surface analysis. Figure 2d shows that a number of byproducts were generated on the bare Zn surface, which is known as ZHS, such as (Zn(OH)_2_)_3_(ZnSO_4_)(H_2_O)_3_ or (Zn(OH)_2_)_3_(ZnSO_4_)(H_2_O)_5_ [35]. The ZHS formed on the electrode can deteriorate cell performance due to its insulating property and continuous water consumption [51]. However, in Figure 2e, some corrosion occurred, but it was confirmed that the pristine surface of Zn was maintained relatively well after removing the PNF layer from PNF-Zn. The FTIR spectra and XRD data further confirmed that the byproducts formed on the Zn surface are ZHS and that PNF has superior corrosion resistance, prohibiting ZHS formation on the surface of PNF-Zn (Figure 3f and Figure S3). In FTIR spectra, the peaks related to H_2_O (O-H stretching vibrations and H_2_O bending vibrations) and SO_4_ (S-O bending vibrations and O-S-O stretching vibrations) were significantly reduced with the PNF-Cu electrode. The XRD data in Figure S3 also showed that the intensity of the ZHS-related peaks at 16.2, 24.4, and 32.7° was substantially suppressed in the PNF layer compared to the bare Zn [52].

### 3.3. Zn Deposition Morphology

After electrodeposition of 5 mAh cm^−2^ Zn on the PNF-Cu electrode under various current densities, the PNF layer was carefully removed to observe the morphology of Zn deposition, and it was confirmed that Zn deposition occurred below the PNF layer (Figure S4).

The SEM and digital images in Figure 3 and Figure S5 show a uniform and compact surface without dendrites with the PNF layer, indicating that the PNF layer effectively suppresses dendrite formation and induces uniform Zn deposition. In contrast, a non-uniform and loose deposition morphology was observed on the bare Cu electrodes. In particular, the difference in Zn deposition morphology between the Cu electrode and the PNF-Cu electrode at a high current density (20 mA cm^−2^) was significant. On the surface of the Cu electrode, sharp dendrites were observed, while a smooth surface was observed on the PNF-Cu electrode. Considering that Zn is easily deposited at the initial nucleation site and protrusion grows, it can be inferred that PNF made the electric field uniform in the PNF-Cu electrode, leading to uniform Zn electrodeposition. To verify the effectiveness of the PNF layer, we constructed a Zn‖Cu half cell with two glass fiber separators that have a fiber morphology similar to that of PNFs but have a higher thickness than that of PNFs and observed a Zn deposition morphology under 1 mA cm^−2^ with a capacity of 5 mAh cm^−2^ (Figure S6). The nonuniform Zn morphology was observed in the cell, as shown in Figure S7, and the result indicates that the PNF layer is not a simple mechanical barrier, and the uniform Zn deposition comes from the high polarity of the PNF layer.

### 3.4. Electrochemical Properties of the Half Cells and Symmetric Cells

**Figure 4:**
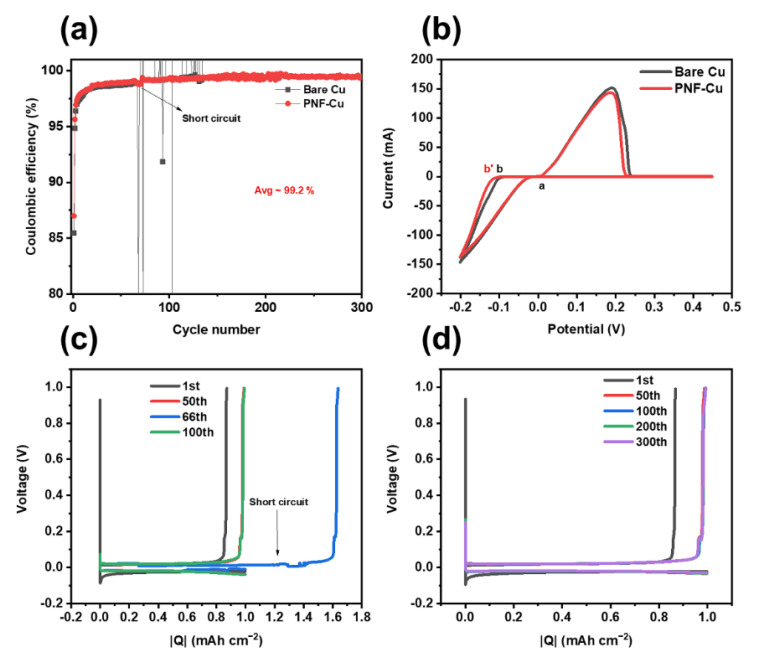
(**a**) Coulombic efficiency, (**b**) cyclic voltammetry, and voltage profile of (**c**) Zn‖Cu half cell and (**d**) Zn‖PNF-Cu half cell.

Zn‖Cu and Zn‖PNF-Cu half cells were constructed to evaluate the reversibility of Zn plating/stripping of the PNF-Cu electrode. The PNF-Cu electrode exhibited a high average coulombic efficiency (CE) of 99.2% over 300 cycles and a stable cycling performance without a short-circuit, while the Zn‖Cu half-cell and Zn‖Cu half-cell specimens using two separators showed a short-circuit from dendrite formation before the 300th cycle under 1 mA cm^−2^ with a capacity of 1 mAh cm^−2^ (Figure 4a,c,d and Figure S8a). These results indicate that when Zn ions are electrodeposited on the Cu electrode surface, dendrites are formed by the localized current density around the tip. In contrast, in the case of the PNF-Cu electrode, the aligned molecular structure of the PNF layer can respond to the localized electric field, relax the localized current density, and inhibits dendrite formation, leading to reversible Zn plating/stripping. The PVDF-Cu electrode was also tested to examine the effect of the β-phase ratio on cell performance, and a short-circuit occurred at approximately 160 cycles, demonstrating poor cycling stability compared to the PNF-Cu electrode (Figure S8b) due to the fact that the fraction of the β-phase of PVDF nanofibers is approximately 10% lower than that of PNF, signifying the importance of the β-phase for performance improvement.

The cyclic voltammetry (CV) test was carried out with the Cu or PNF-Cu and Zn electrodes as working and counter electrodes, respectively (Figure 4b). This showed that the shape of the CV graph was well-maintained after the introduction of the nanofiber layer, so the PNF layer remained stable during battery operation and did not affect the reversible Zn/Zn^2+^ reaction. A crossover point (a) can be observed in the positive potential sweeping. The PNF-Cu electrode showed a slightly higher nucleation overpotential (a-b) than the bare Cu electrode (a-b′) due to the hydrophobic nature of the PNF layer.

**Figure 5:**
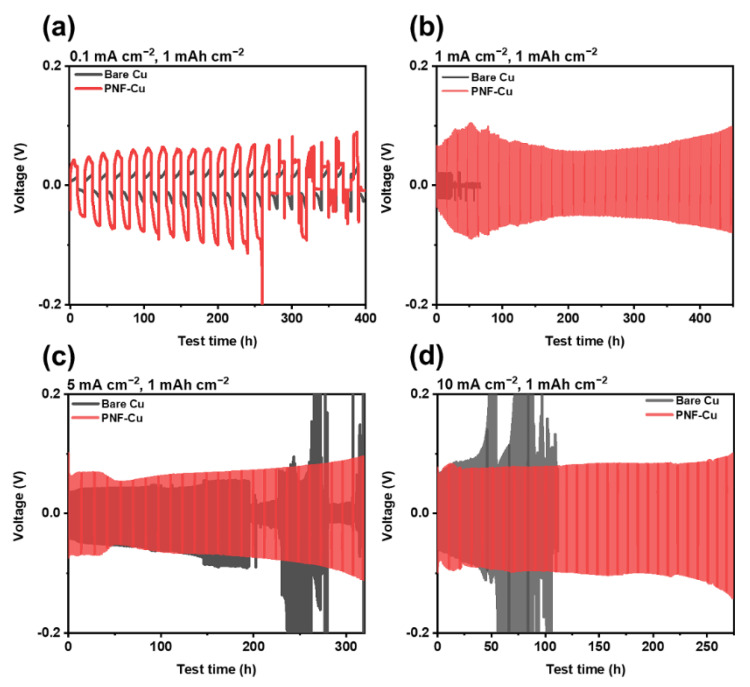
(**a**) Voltage–time curves of symmetric cells of zinc-deposited bare copper and P(VDF-TrFE) electrospun Cu at (**a**) 0.1, (**b**) 1, (**c**) 5, and (**d**) 10 mA cm^−2^.

The electrochemical stability of the PNF-Cu anode was further investigated by galvanostatic cycling of the symmetric cell under diverse current densities (Figure 5). Before the cell assembly, 5 mAh cm^−2^ Zn was electrodeposited on the Cu electrode and the PNF-Cu electrode at a rate of 1 mA cm^−2^. Under 1–10 mA cm^−2^, the PNF-Cu showed stable plating/stripping cycles with a steady voltage–time profile but high overpotential derived from the hydrophobicity of the PNF layer. It is noticeable that at a current density of 0.1 mA cm^−2^, the PNF-Cu symmetric cell showed a shorter lifecycle than that of the bare Cu symmetric cell. The shorter lifecycle of the symmetric cell using Zn@PNF-Cu indicates that the low current density of 0.1 mA cm^−2^ is not sufficient to make oriented dipole moments along the external electric field.

This result implies that a moderate current density is required to utilize the PNF layer, and further research to make PNF hydrophilic is required to utilize the PNF layer to reduce the overpotential of the cell. The voltage–time profiles in Figure 5a–d show that as the current density increased, the overpotential of the Zn@Cu symmetric cells during cycling dramatically increased due to nonuniform dendritic Zn growth. However, in the case of the Zn@PNF-Cu symmetric cell, it was confirmed that the increase in the overpotential by the current density was not significant compared to the Zn@Cu symmetric cell due to uniform Zn deposition, although it showed a larger overpotential than the Zn@Cu symmetric cell, ascribed to the hydrophobic nature of PNF (Figure 5a–d). As a result, at a high current density of 10 mA cm^−2^, the Zn@PNF-Cu symmetric cell maintained a stable voltage profile over 250 h, while Zn@Cu symmetric cells failed only after 40 h with increasing voltage hysteresis. Thus, the results imply that the PNF layer can suppress dendrite formation for a longer time without short-circuiting under various current densities.

### 3.5. Full-Cell Test

**Figure 6:**
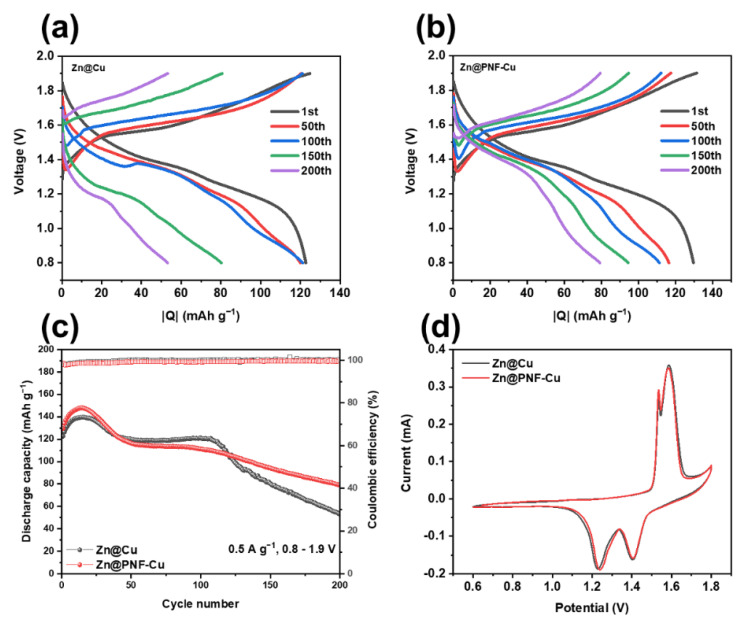
Zn-MnO_2_ full-cell performance: charge and discharge curves of the cells using (**a**) Zn@Cu and (**b**) Zn@PNF-Cu, (**c**) cycling performance at 0.5 mA g^−1^, and (**d**) CV curves at a scan rate of 0.1 mV s^−1^.

To validate the practical use of the PNF-Cu electrode as an anode material for aqueous ZIBs, a full-cell performance test was conducted after pairing it with a MnO_2_ cathode. Figure 6a presents the charge–discharge profiles of the Zn@Cu cell. The voltage gap rapidly increased, and the capacity decreased as the cycle progressed. However, in the cell using the Zn@PNF-Cu electrode (Figure 6b), the increase in the voltage gap and the decrease in capacity during repeated cycling were restrained. At a current density of 0.5 A g^−1^, the cell using the Zn@PNF-Cu electrode showed moderate capacity fading during cycling, delivering 79.18 mAh g^−1^ after 200 cycles, whereas the cell using the Zn@Cu electrode showed rapid capacity fading after 120 cycles, delivering only 53.16 mAh g^−1^ after 200 cycles (Figure 6c). This is because when a Zn@PNF-Cu electrode is used, reversible Zn plating/stripping occurs, and side reactions between the electrode interfaces can be curbed.

Additionally, the CE in the case of Zn@Cu often exceeds 100%, which seems to be due to a side reaction between the electrode and the electrolyte interface, and this phenomenon is not observed in the Zn@PNF-Cu electrode system. The CV curves are shown in Figure 6d, illustrating the high reversibility of the full cells. Both electrodes displayed the two characteristic redox peaks of MnO_2_ ascribed to multiple phase conversions with similar peak currents and positions, confirming that the PNF layer on the anode does not affect the reversible electrochemical reactions of the MnO_2_ cathode [52,53,54]. The performance of the electrospun Zn@PNF-Cu anode is superior to many previous works, as summarized in Table S1. Some of the previous zinc anodes presented excellent cycling stability under low current density, whereas some anodes worked at higher current density, and they could only deliver a limited number of cycles. These promising results verify the remarkable electrochemical performance and possible use in practical applications of the Zn@PNF-Cu electrode in ZIBs.

## 4. Conclusions

In summary, we introduced electrospun PNF onto a Cu electrode to address the dendrite formation issue in ZIBs. We confirmed that a high proportion of β-crystalline phase was formed in PNFs through electrospinning followed by heat treatment. After the introduction of the PNF layer, the formation of dendrites was effectively suppressed by alleviating the local current density with its unique aligned molecular structure. Moreover, ascribed to its hydrophobic properties, the PNF layer can suppress the side reaction between the electrode and electrolyte. As a result, the PNF-Cu‖Zn half-cell configuration achieved an average coulombic efficiency of 99.2% over 300 cycles. Additionally, the possibility of practical use was confirmed by maintaining cycling stability for 200 cycles at 0.5 A g^−1^ when a full cell was constructed with the MnO_2_ cathode. Based on the above results, we anticipate that the PNF-Cu electrode can provide a practical and meaningful approach to safe and highly efficient ZIBs.

## Data Availability

Not applicable.

## Supplementary Materials

The following supporting information can be downloaded at: https://www.mdpi.com/article/10.3390/membranes12101014/s1: Figure S1: The voltage-time curves during Zinc nucleation at 1 mA cm^−2^ on Zn and Cu substrate; Figure S2: Ionic resistivity measurement of the bare Cu and PNF-Cu symmetric cells with the glass fiber as a separator. Nyquist plots were tested at open circuit voltage (OCV) over the frequency range of 100 kHz to 0.1 Hz; Figure S3: XRD data of the bare Zn and PNF-Zn after immersion in the electrolyte for 7 days; Figure S4: Digital images of Zn deposition on the (a) bare Cu and PNF-Cu (b) before removal and (c) after removal of the PNF layer; Figure S5: Top-view SEM images of Zn deposition morphology on (a-d) bare Cu electrode and (e-h) PNF-Cu under various current densities; Figure S6: Top-view SEM image of glass fiber; Figure S7: Top-view SEM images of Zn deposition morphology of Zn‖Cu cell using two glass fibers as a separator; Figure S8: Coulombic efficiencies of long-term cycles at 1 mA cm^−^^2^ of the (a) Zn‖Cu half cell using 2 separators and (b) Zn‖PVDF-Cu half cell; Table S1: Comparison table for the electrochemical performances by various materials used for surface modification of Zn anode.and PNF-Cu under various current densities. References [55,56,57,58,59,60,61,62,63,64,65,66,67,68,69,70,71,72,73] are cited in the Supplementary Materials.

## References

[1] Kim J.-K., Mueller F., Kim H., Jeong S., Park J.-S., Passerini S., Kim Y. (2016). Eco-friendly Energy Storage System: Seawater and Ionic Liquid Electrolyte. ChemSusChem.

[2] Alam R.B., Ahmad M.H., Islam M.R. (2021). Bio-inspired gelatin/single-walled carbon nanotube nanocomposite for transient electrochemical energy storage: An approach towards eco-friendly and sustainable energy system. Heliyon.

[3] Chang N., Li T., Li R., Wang S., Yin Y., Zhang H., Li X. (2020). An aqueous hybrid electrolyte for low-temperature zinc-based energy storage devices. Energy Environ. Sci..

[4] Mensah-Darkwa K., Zequine C., Kahol P.K., Gupta R.K. (2019). Supercapacitor Energy Storage Device Using Biowastes: A Sustainable Approach to Green Energy. Sustainability.

[5] Choi J.W., Aurbach D. (2016). Promise and reality of post-lithium-ion batteries with high energy densities. Nat. Rev. Mater..

[6] Ogumi Z., Kostecki R., Guyomard D., Inaba M. (2016). Lithium-Ion Batteries-The 25th Anniversary of Commercialization. Electrochem. Soc. Interface.

[7] Yoshino A. (2012). The Birth of the Lithium-Ion Battery. Angew. Chem. Int. Ed..

[8] Liu J., Xu C., Chen Z., Ni S., Shen Z.X. (2018). Progress in aqueous rechargeable batteries. Green Energy Environ..

[9] Balakrishnan P.G., Ramesh R., Prem Kumar T. (2006). Safety mechanisms in lithium-ion batteries. J. Power Sources.

[10] Chawla N., Bharti N., Singh S. (2019). Recent Advances in Non-Flammable Electrolytes for Safer Lithium-Ion Batteries. Batteries.

[11] Wang F., Borodin O., Ding M.S., Gobet M., Vatamanu J., Fan X., Gao T., Eidson N., Liang Y., Sun W. (2018). Hybrid Aqueous/Non-aqueous Electrolyte for Safe and High-Energy Li-Ion Batteries. Joule.

[12] Dou Q., Wang Y., Wang A., Ye M., Hou R., Lu Y., Su L., Shi S., Zhang H., Yan X. (2020). Water in salt/ionic liquid” electrolyte for 2.8 V aqueous lithium-ion capacitor. Sci. Bull..

[13] Ren J., Li C., Li H., Li Z., Liu S., Luo B., Wang L. (2022). Realizing highly stable zinc-ion batteries via electrolyte engineering with adsorbed molecular protective layer. Electrochim. Acta.

[14] Sun P., Liu W., Yang D., Zhang Y., Xiong W., Li S., Chen J., Tian J., Zhang L. (2022). Stable Zn anodes enabled by high-modulus agarose gel electrolyte with confined water molecule mobility. Electrochim. Acta.

[15] Hoang Huy V.P., Hieu L.T., Hur J. (2021). Zn Metal Anodes for Zn-Ion Batteries in Mild Aqueous Electrolytes: Challenges and Strategies. Nanomaterials.

[16] Yan J., Ang E.H., Yang Y., Zhang Y., Ye M., Du W., Li C.C. (2021). High-Voltage Zinc-Ion Batteries: Design Strategies and Challenges. Adv. Funct. Mater..

[17] Chen D., Lu M., Cai D., Yang H., Han W. (2021). Recent advances in energy storage mechanism of aqueous zinc-ion batteries. J. Energy Chem..

[18] Kim E., Choi I., Nam K.W. (2022). Metal–organic framework for dendrite-free anodes in aqueous rechargeable zinc batteries. Electrochim. Acta.

[19] Yang Q., Li Q., Liu Z., Wang D., Guo Y., Li X., Tang Y., Li H., Dong B., Zhi C. (2020). Dendrites in Zn-Based Batteries. Adv. Mater..

[20] Kumar S., Yoon H., Park H., Park G., Suh S., Kim H.-J. (2022). A dendrite-free anode for stable aqueous rechargeable zinc-ion batteries. J. Ind. Eng. Chem..

[21] Liu B., Wang S., Wang Z., Lei H., Chen Z., Mai W. (2020). Novel 3D Nanoporous Zn–Cu Alloy as Long-Life Anode toward High-Voltage Double Electrolyte Aqueous Zinc-Ion Batteries. Small.

[22] Li B., Xue J., Han C., Liu N., Ma K., Zhang R., Wu X., Dai L., Wang L., He Z. (2021). A hafnium oxide-coated dendrite-free zinc anode for rechargeable aqueous zinc-ion batteries. J. Colloid Interface Sci..

[23] Chen P., Yuan X., Xia Y., Zhang Y., Fu L., Liu L., Yu N., Huang Q., Wang B., Hu X. (2021). An Artificial Polyacrylonitrile Coating Layer Confining Zinc Dendrite Growth for Highly Reversible Aqueous Zinc-Based Batteries. Adv. Sci..

[24] Jian Q., Wan Y., Sun J., Wu M., Zhao T. (2020). A dendrite-free zinc anode for rechargeable aqueous batteries. J. Mater. Chem..

[25] He H., Qin H., Wu J., Chen X., Huang R., Shen F., Wu Z., Chen G., Yin S., Liu J. (2021). Engineering interfacial layers to enable Zn metal anodes for aqueous zinc-ion batteries. Energy Stor. Mater..

[26] Pusty M., Shirage P.M. (2022). Insights and perspectives on graphene-PVDF based nanocomposite materials for harvesting mechanical energy. J. Alloys Compd..

[27] Yu Y., Shao W., Zhong J., Ye H., Yang L., Zhen L. (2021). Tuning the Energy Storage Efficiency in PVDF Nanocomposites Incorporated with Crumpled Core–Shell BaTiO3@Graphene Oxide Nanoparticles. ACS Appl. Energy Mater..

[28] Zhang M., Tan S., Xiong J., Chen C., Zhang Y., Wei X., Zhang Z. (2021). Tailoring Dielectric and Energy Storage Performance of PVDF-Based Relaxor Ferroelectrics with Hydrogen Bonds. ACS Appl. Energy Mater..

[29] Yi Z., Wang Z., Nian W., Wang T., Chen H., Cheng Z. (2021). High Energy Storage Density of Sandwich-Structured Na_0.5_Bi_0.5_TiO_3_/PVDF Nanocomposites Enhanced by Optimizing the Dimensions of Fillers. ACS Appl. Energy Mater..

[30] Shepelin N.A., Sherrell P.C., Skountzos E.N., Goudeli E., Zhang J., Lussini V.C., Imtiaz B., Usman K.A.S., Dicinosk G.W., Shapter J.G. (2021). Interfacial piezoelectric polarization locking in printable Ti_3_C_2_T_x_ MXene-fluoropolymer composites. Nat. Commun..

[31] Fortunato M., Cavallini D., Bellis G.D., Marra F.A., Tamburrano F., Sarto M.S., Sarto F. (2019). Phase Inversion in PVDF Films with Enhanced Piezoresponse Through Spin-Coating and Quenching. Polymers.

[32] Kim M., Lee S., Kim Y.-I. (2020). Solvent-controlled crystalline beta-phase formation in electrospun P(VDF-TrFE) fibers for enhanced piezoelectric energy harvesting. APL Mater..

[33] Song W.-J., Joo S.H., Kim D.H., Hwang C., Jung G.Y., Bae S., Son Y., Cho J., Song H.-K., Kwak S.K. (2017). Significance of ferroelectric polarization in poly (vinylidene difluoride) binder for high-rate Li-ion diffusion. Nano Energy.

[34] Hwang C., Song W.-J., Song G., Wu Y., Lee S., Son H.B., Kim A.J., Liu N., Park S., Song H.-K. (2020). A Three-Dimensional Nano-web Scaffold of Ferroelectric Beta-PVDF Fibers for Lithium Metal Plating and Stripping. ACS Appl. Mater. Interfaces.

[35] Wang Y., Liu Y., Wang H., Dou S., Gan W., Ci L., Huang Y., Yuan Q. (2022). MOF-based ionic sieve interphase for regulated Zn^2+^ flux toward dendrite-free aqueous zinc-ion batteries. J. Mater. Chem..

[36] Zhang C., Zhang T., Feng M., Cui Y., Zhang T., Zhang Y., Feng Y., Zhang Y., Chi Q., Liu X. (2021). Significantly Improved Energy Storage Performance of PVDF Ferroelectric Films by Blending PMMA and Filling PCBM. ACS Sustain. Chem. Eng..

[37] Mayeen A., Kala M.S., Sunija S., Rouxel D., Bhowmik R.N., Thomas S., Kalarikkal N. (2020). Flexible dopamine-functionalized BaTiO_3_/BaTiZrO_3_/BaZrO_3_-PVDF ferroelectric nanofibers for electrical energy storage. J. Alloys Compd..

[38] Wang Y., Guo T., Yin J., Tian Z., Ma Y., Liu Z., Zhu Y., Alshareef H.N. (2020). Controlled Deposition of Zinc-Metal Anodes via Selectively Polarized Ferroelectric Polymers. Adv. Mater..

[39] Mai M., Ke S., Lin P., Zeng X. (2015). Ferroelectric Polymer Thin Films for Organic Electronics. J. Nanomater..

[40] Abrha L.H., Nikodimos Y., Weldeyohannes H.H., Hagos T.T., Wang D.-Y., Huang C.-J., Jiang S.-K., Wu S.-H., Su W.-N., Tsai M.-C. (2021). Effects of a Thermally Electrochemically Activated β-PVDF Fiber on Suppression of Li Dendrite Growth for Anode-Free Batteries. ACS Appl. Energy Mater..

[41] Li C., Qiu M., Li R., Li X., Wang M., He J., Lin G., Xiao L., Qian Q., Chen Q. (2022). Electrospinning Engineering Enables High-Performance Sodium-Ion Batteries. Adv. Fiber Mater..

[42] Li R., Wu J., He J., Li X., Mai Y., Chen Y., Li X. (2022). Embedding amorphous SnS in electrospun porous carbon nanofibers for efficient potassium storage with ultralong cycle life. Compos. B Eng..

[43] Li X., Chen W., Qian Q., Huang H., Chen Y., Wang Z., Chen Q., Yang J., Li J., Mai Y. (2021). Electrospinning-Based Strategies for Battery Materials. Adv. Energy Mater..

[44] Xie S., Li Y., Li X., Zhou Y., Dang Z., Rong J., Dong L. (2021). Stable Zinc Anodes Enabled by Zincophilic Cu Nanowire Networks. Nanomicro Lett.

[45] Zhou L., Yang F., Zeng S., Gao X., Liu X., Cao X., Yu P., Lu X. (2022). Zincophilic Cu Sites Induce Dendrite-Free Zn Anodes for Robust Alkaline/Neutral Aqueous Batteries. Adv. Funct. Mater..

[46] Xie C., Li Y., Wang Q., Sun D., Tang T., Wang H. (2020). Issues and solutions toward zinc anode in aqueous zinc-ion batteries: A mini review. Carbon Energy.

[47] Ruan L., Yao X., Chang Y., Zhou L., Qin G., Zhang X. (2018). Properties and Applications of the β Phase Poly(vinylidene fluoride). Polymers.

[48] Satthiyaraju M., Ramesh T. (2019). Effect of annealing treatment on PVDF nanofibers for mechanical energy harvesting applications. Mater. Res. Express.

[49] Poudel A., Fernandez M.A., Tofail S.A.M., Biggs M.J.P. (2019). Boron Nitride Nanotube Addition Enhances the Crystallinity and Cytocompatibility of PVDF-TrFE. Front. Chem..

[50] Wu Y., Du X., Gao R., Li J., Li W., Yu H., Jiang Z., Wang Z., Tai H. (2019). Self-Polarization of PVDF Film Triggered by Hydrophilic Treatment for Pyroelectric Sensor with Ultra-Low Piezoelectric Noise. Nanoscale Res. Lett..

[51] Zhang Q., Yang Z., Ji H., Zeng X., Tang Y., Sun D., Wang H. (2021). Issues and rational design of aqueous electrolyte for Zn-ion batteries. SusMat.

[52] Yang J., Cao J., Peng Y., Yang W., Barg S., Liu Z., Kinloch I.A., Bissett M.A., Dryfe R.A.W. (2020). Unravelling the Mechanism of Rechargeable Aqueous Zn–MnO_2_ Batteries: Implementation of Charging Process by Electrodeposition of MnO_2_. ChemSusChem.

[53] Li L., Hoang T.K.A., Zhi J., Han M., Li S., Chen P. (2020). Functioning Mechanism of the Secondary Aqueous Zn-β-MnO_2_ Battery. ACS Appl. Mater. Interfaces.

[54] Liu W., Zhang X., Huang Y., Jiang B., Chang Z., Xu C., Kang F. (2021). β-MnO_2_ with proton conversion mechanism in rechargeable zinc ion battery. J. Energy Chem..

[55] Cui Y., Zhao Q., Wu X., Chen X., Yang J., Wang Y., Qin R., Ding S., Song Y., Wu J. (2020). An Interface-Bridged Organic–Inorganic Layer That Suppresses Dendrite Formation and Side Reactions for Ultra-Long-Life Aqueous Zinc Metal Anodes. Angew. Chem..

[56] Cui M., Xiao Y., Kang L., Du W., Gao Y., Sun X., Zhou Y., Li X., Li H., Jiang F. (2019). Quasi-Isolated Au Particles as Heterogeneous Seeds to Guide Uniform Zn Deposition for Aqueous Zinc-Ion Batteries. ACS Appl. Energy Mater..

[57] Zhao Z., Zhao J., Hu Z., Li J., Li J., Zhang Y., Wang C., Cui G. (2019). Long-Life and Deeply Rechargeable Aqueous Zn Anodes Enabled by a Multifunctional Brightener-Inspired Interphase. Energy Environ. Sci..

[58] Cai Z., Ou Y., Wang J., Xiao R., Fu L., Yuan Z., Zhan R., Sun Y. (2020). Chemically Resistant Cu–Zn/Zn Composite Anode for Long Cycling Aqueous Batteries. Energy Storage Mater..

[59] Hieu L.T., So S., Kim I.T., Hur J. (2021). Zn Anode with Flexible β-PVDF Coating for Aqueous Zn-Ion Batteries with Long Cycle Life. Chem. Eng. J..

[60] Zhou M., Guo S., Fang G., Sun H., Cao X., Zhou J., Pan A., Liang S. (2021). Suppressing by-Product via Stratified Adsorption Effect to Assist Highly Reversible Zinc Anode in Aqueous Electrolyte. J. Energy Chem..

[61] Xie X., Liang S., Gao J., Guo S., Guo J., Wang C., Xu G., Wu X., Chen G., Zhou J. (2020). Manipulating the Ion-Transfer Kinetics and Interface Stability for High-Performance Zinc Metal Anodes. Energy Environ. Sci..

[62] Cao Z., Zhu X., Xu D., Dong P., Chee M.O., Li X., Zhu K., Ye M., Shen J. (2021). Eliminating Zn Dendrites by Commercial Cyanoacrylate Adhesive for Zinc Ion Battery. Energy Storage Mater..

[63] Yang Y., Liu C., Lv Z., Yang H., Zhang Y., Ye M., Chen L., Zhao J., Li C.C. (2021). Synergistic Manipulation of Zn^2+^ Ion Flux and Desolvation Effect Enabled by Anodic Growth of a 3D ZnF_2_ Matrix for Long-Lifespan and Dendrite-Free Zn Metal Anodes. Adv. Mater..

[64] Miao Z., Du M., Li H., Zhang F., Jiang H., Sang Y., Li Q., Liu H., Wang S. (2021). Constructing Nano-Channeled Tin Layer on Metal Zinc for High-Performance Zinc-Ion Batteries Anode. EcoMat.

[65] Ma C., Wang X., Lu W., Wang C., Yue H., Sun G., Zhang D., Du F. (2022). Achieving Stable Zn Metal Anode via a Simple Nico Layered Double Hydroxides Artificial Coating for High Performance Aqueous Zn-Ion Batteries. Chem. Eng. J..

[66] Yang Y., Liu C., Lv Z., Yang H., Cheng X., Zhang S., Ye M., Zhang Y., Chen L., Zhao J. (2021). Redistributing Zn-Ion Flux by Interlayer Ion Channels in MG-Al Layered Double Hydroxide-Based Artificial Solid Electrolyte Interface for Ultra-Stable and Dendrite-Free Zn Metal Anodes. Energy Storage Mater..

[67] Guo Z., Fan L., Zhao C., Chen A., Liu N., Zhang Y., Zhang N. (2021). A Dynamic and Self-Adapting Interface Coating for Stable Zn-Metal Anodes. Adv. Mater..

[68] Cao J., Zhang D., Gu C., Wang X., Wang S., Zhang X., Qin J., Wu Z.S. (2021). Manipulating Crystallographic Orientation of Zinc Deposition for Dendrite-Free Zinc Ion Batteries. Adv. Energy Mater..

[69] Wu C., Xie K., Ren K., Yang S., Wang Q. (2020). Dendrite-Free Zn Anodes Enabled by Functional Nitrogen-Doped Carbon Protective Layers for Aqueous Zinc-Ion Batteries. Dalton Trans..

[70] Zou P., Zhang R., Yao L., Qin J., Kisslinger K., Zhuang H., Xin H.L. (2021). Ultrahigh-Rate and Long-Life Zinc–Metal Anodes Enabled by Self-Accelerated Cation Migration. Adv. Energy Mater..

[71] Cao P., Zhou X., Wei A., Meng Q., Ye H., Liu W., Tang J., Yang J. (2021). Fast-Charging and Ultrahigh-Capacity Zinc Metal Anode for High-Performance Aqueous Zinc-Ion Batteries. Adv. Funct. Mater..

[72] Hao J., Li B., Li X., Zeng X., Zhang S., Yang F., Liu S., Li D., Wu C., Guo Z. (2020). An in-Depth Study of Zn Metal Surface Chemistry for Advanced Aqueous Zn-Ion Batteries. Adv. Mater..

[73] Zhang X., Li J., Liu D., Liu M., Zhou T., Qi K., Shi L., Zhu Y., Qian Y. (2021). Ultra-Long-Life and Highly Reversible Zn Metal Anodes Enabled by a desolvation and deanionization Interface Layer. Energy Environ. Sci..

