# Supplementary material for: Inhibition of Zinc Dendrites Realized by a β-P(VDF-TrFE) Nanofiber Layer in Aqueous Zn-Ion Batteries
Source: Membranes (Basel). 2022 Oct 19;12(10):1014. doi: 10.3390/membranes12101014 (PMC9610699; doi:10.3390/membranes12101014)
Supplement: Supplementary file 1 [file membranes-12-01014-s001.zip › membranes-1943174-supplementary.pdf]

Supporting Information for

Inhibition of Zinc Dendrites Realized by a β-P(VDF-TrFE) Nanofiber Layer in Aqueous Zn-Ion Batteries

Geumyong Park [1,†], Hyeonghun Park [1,†], WooJun Seol [2], Seokho Suh [1], Ji Young Jo [2], Santosh Kumar [1,3,*] and Hyeong-Jin Kim [1,*]

[1] Graduate School of Energy Convergence, Institute of Integrated Technology, Gwangju Institute of Science and Technology (GIST), 123 Cheomdangwagi-ro, Buk-gu, Gwangju 61005, Korea

[2] School of Materials Science and Engineering, Gwangju Institute of Science and Technology (GIST), 123 Cheomdangwagi-ro, Buk-gu, Gwangju 61005, Korea

[3] Research Institute for Solar and Sustainable Energies (RISE), Gwangju Institute of Science and Technology (GIST), 123 Cheomdangwagi-ro, Buk-gu, Gwangju 61005, Korea

* Correspondence: skumar@gist.ac.kr (S.K.); hjkimc@gist.ac.kr (H.-J.K.)

† These authors contributed equally to this work.

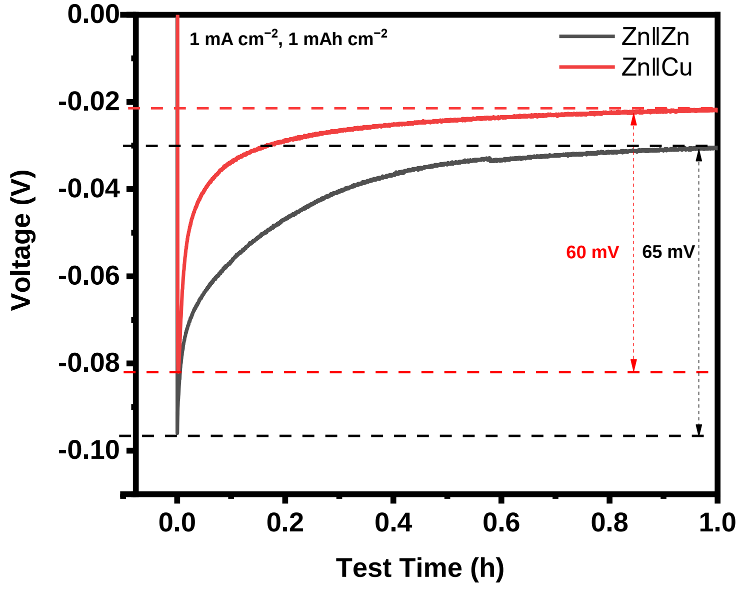

Figure S1. The voltage-time curves during Zinc nucleation at 1 mA cm^{-2} on Zn and Cu substrate.

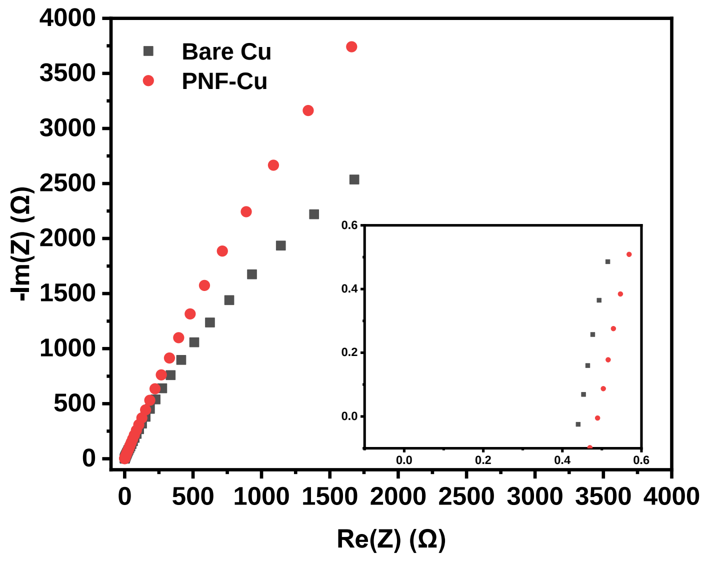

Figure S2. Ionic resistivity measurement of the bare Cu and PNF-Cu symmetric cells with the glass fiber as a separator. Nyquist plots were tested at open circuit voltage (OCV) over the frequency range of 100 kHz to 0.1 Hz.

Formula used for calculating the conductivity,

$$\sigma = \frac{L}{R\mathrm{b}S}$$

Where,

$L_{\text{glass fiber}} = 0.211$ mm

$S = 0.7854$ cm^2

R_b (glass fiber) = 0.4868 Ω

$\sigma_{\text{glass fiber}} = 5.5\times10^{-2}$ S cm^{-2}

R_b(PVDF-TrFE) = R_b-R_b (glass fiber) = 0.04921 Ω

$L_{P(VDF-TrFE)} = 5\ \mu m \times 2 = 10\ \mu m$

$\sigma_{P(VDF-TrFE)} = 2.59\times10^{-2}\ S\ cm^{-2}$

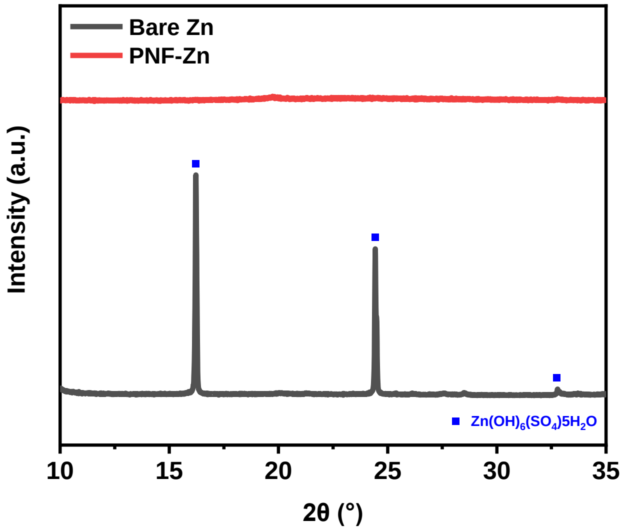

Figure S3. XRD data of the bare Zn and PNF-Zn after immersion in the electrolyte for 7 days.

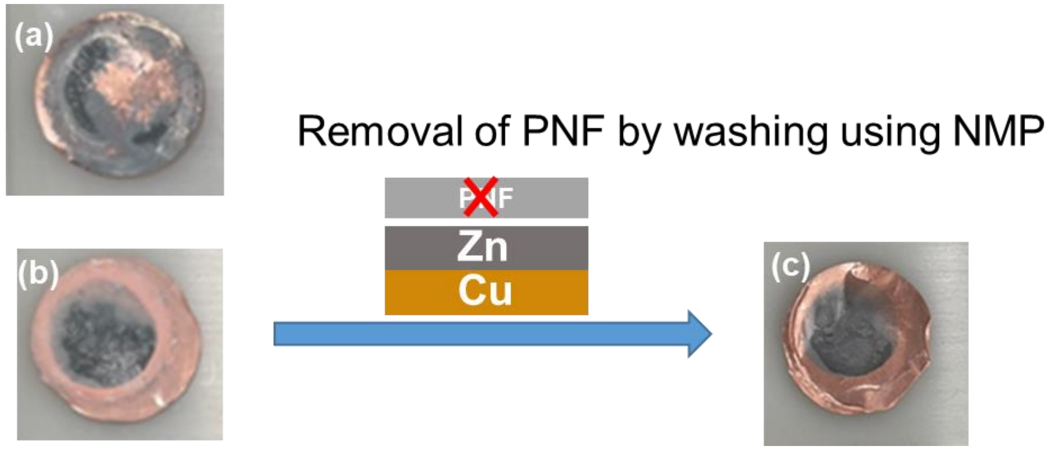

Figure S4. Digital images of Zn deposition on the (a) bare Cu and PNF-Cu (b) before removal and (c) after removal of the PNF layer.

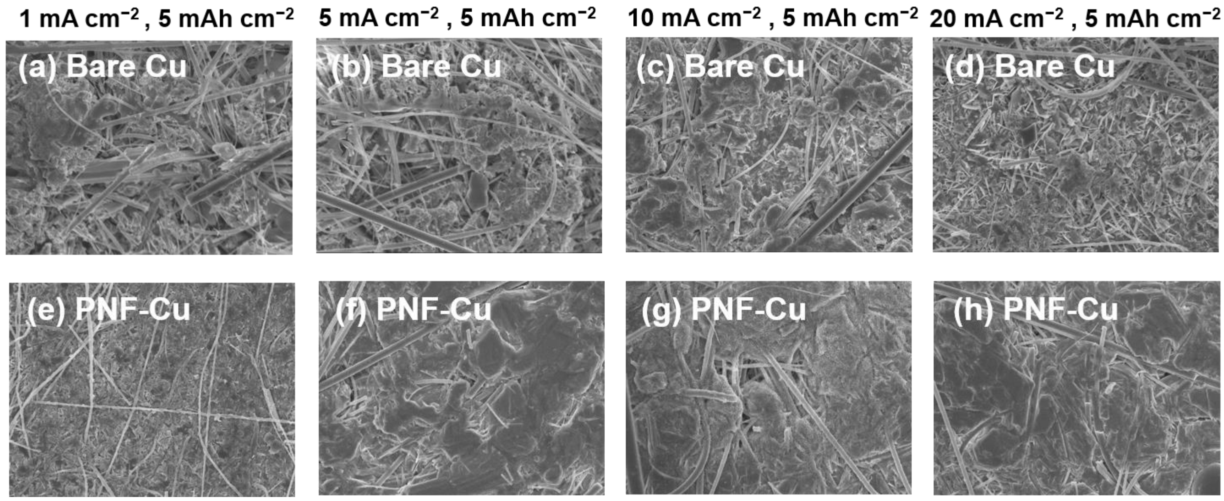

Figure S5. Top-view SEM images of Zn deposition morphology on (a-d) bare Cu electrode and (e-h) PNF-Cu under various current densities.

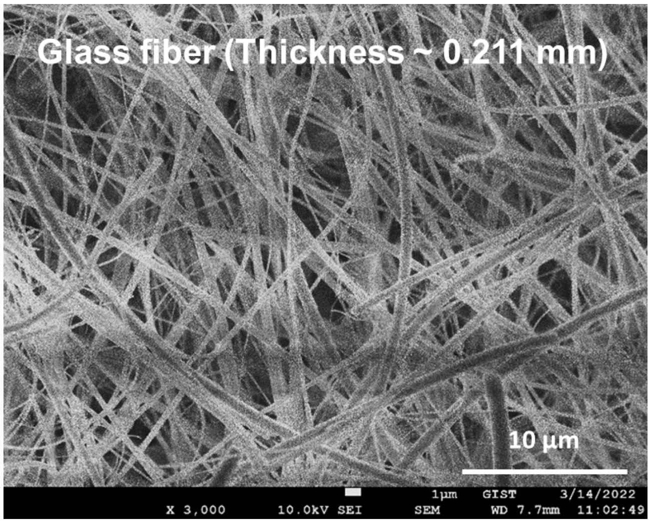

Figure S6. Top-view SEM image of glass fiber.

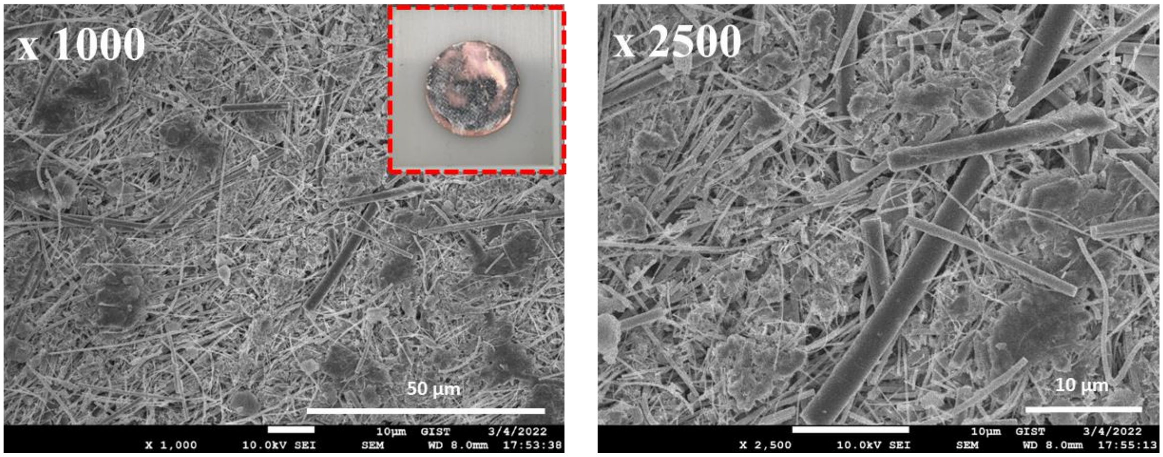

Figure S7. Top-view SEM images of Zn deposition morphology of Zn‖Cu cell using two glass fibers as a separator.

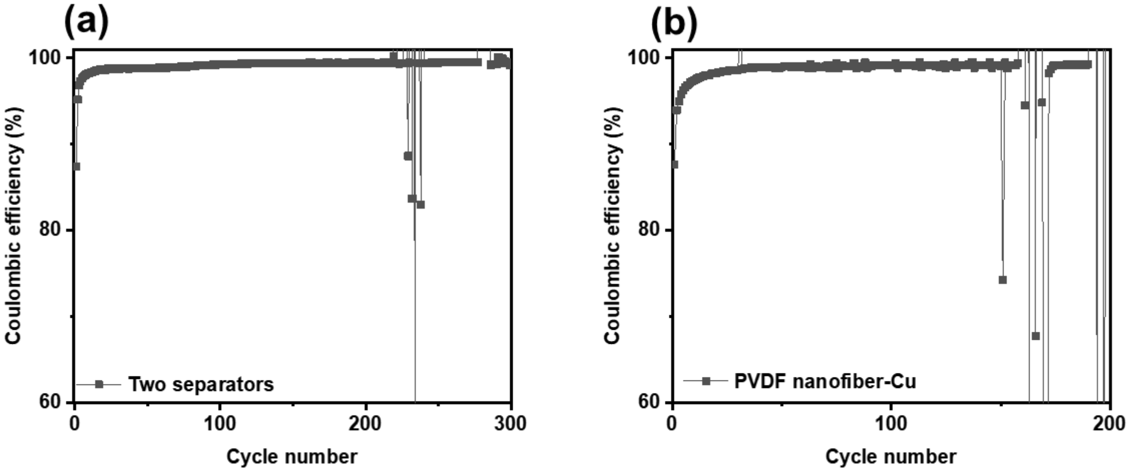

Figure S8. Coulombic efficiencies of long-term cycles at 1 mA cm^{-2} of the (a) Zn‖Cu half cell using 2 separators and (b) Zn‖PVDF-Cu half cell.

Table S1. Comparison table for the electrochemical performances by various materials used for surface modification of Zn anode.

Materials	Areal capacity (mA h cm^{-2})	Current density (mA cm^{-2})	Coulombic Efficiency (%)	Cycle number	Reference
PNF	1	1	99.2	300	Present work
Nafion Zn-X	2	0.2	97	130	1
nanoAu	0.5	0.5	97.1	60	2
PA layer[a]	0.4	0.4	97.43	300	3
Cu/Zn	0.5	5	91.8	100	4
β-PVDF	0.18	0.36	~ 96.5	200	5
Sc_2O_3	0.56	1.13	99.85	260	6
ZnO	0.5	2	99.55	300	7
502 glue	1	2	99.74	200	8
ZnF_2	1	5	99.5	1000	9
Sn	1	1	~ 99	250	10
NCLTi[b]	1	2	99.0	~700	11
Mg-Al LDH[c]	1	10	99.2	2000	12
PDMS/TiO_{2-x}[d]	0.5	0.5	~ 99.4	450	13
CG separator[e]	1	1	98.68	100	14
N-C[f]	2	2	98.76	120	15
$BaTiO_3$	1	1	/	120	16
ZnP	0.5	2	99.5	200	17
ZnS	1	2	99.2	200	18
CNG[g]	1	0.5	99.4	300	19

[a]Polyamide (PA), [b]NiCo layered double hydroxides (NCLTi), [c]Mg-Al layered double hydroxide (Mg-Al layered double hydroxide), [d]Poly(dimethylsiloxane) (PDMS), [e]Cellulose nanofibers and graphene oxide (CG), [f]Conductive and defective N-doped carbon (N-C), [g]Celluose nanowhisker-graphene (CNG).

References

1. Cui, Y.; Zhao, Q.; Wu, X.; Chen, X.; Yang, J.; Wang, Y.; Qin, R.; Ding, S.; Song, Y.; Wu, J.; Yang, K.; Wang, Z.; Mei, Z.; Song, Z.; Wu, H.; Jiang, Z.; Qian, G.; Yang, L.; Pan, F. An Interface-Bridged Organic–Inorganic Layer That Suppresses Dendrite Formation and Side Reactions for Ultra-Long-Life Aqueous Zinc Metal Anodes. *Angew. Chem.* **2020**, 16737-16744.

2. Cui, M.; Xiao, Y.; Kang, L.; Du, W.; Gao, Y.; Sun, X.; Zhou, Y.; Li, X.; Li, H.; Jiang, F.; Zhi, C. Quasi-Isolated Au Particles as Heterogeneous Seeds to Guide Uniform Zn Deposition for Aqueous Zinc-Ion Batteries. *ACS Appl. Energy Mater.* **2019**, *2*, 6490–6496.

3. Zhao, Z.; Zhao, J.; Hu, Z.; Li, J.; Li, J.; Zhang, Y.; Wang, C.; Cui, G. Long-Life and Deeply Rechargeable Aqueous Zn Anodes Enabled by a Multifunctional Brightener-Inspired Interphase. *Energy Environ. Sci.* **2019**, *12*, 1938–1949.

4. Cai, Z.; Ou, Y.; Wang, J.; Xiao, R.; Fu, L.; Yuan, Z.; Zhan, R.; Sun, Y. Chemically Resistant Cu–Zn/Zn Composite Anode for Long Cycling Aqueous Batteries. *Energy Storage Mater.* **2020**, *27*, 205–211.

5. Hieu, L. T.; So, S.; Kim, I. T.; Hur, J. Zn Anode with Flexible β-PVDF Coating for Aqueous Zn-Ion Batteries with Long Cycle Life. *Chem. Eng. J.* **2021**, *411*, 128584.

6. Zhou, M.; Guo, S.; Fang, G.; Sun, H.; Cao, X.; Zhou, J.; Pan, A.; Liang, S. Suppressing by-Product via Stratified Adsorption Effect to Assist Highly Reversible Zinc Anode in Aqueous *Electrolyte*. *J. Energy Chem.* **2021**, *55*, 549–556.

7. Xie, X.; Liang, S.; Gao, J.; Guo, S.; Guo, J.; Wang, C.; Xu, G.; Wu, X.; Chen, G.; Zhou, J. Manipulating the Ion-Transfer Kinetics and Interface Stability for High-Performance Zinc Metal Anodes. *Energy Environ. Sci.* **2020**, *13*, 503–510.

8. Cao, Z.; Zhu, X.; Xu, D.; Dong, P.; Chee, M. O.; Li, X.; Zhu, K.; Ye, M.; Shen, J. Eliminating Zn Dendrites by Commercial Cyanoacrylate Adhesive for Zinc Ion Battery. *Energy Storage Mater.* **2021**, *36*, 132–138.

9. Yang, Y.; Liu, C.; Lv, Z.; Yang, H.; Zhang, Y.; Ye, M.; Chen, L.; Zhao, J.; Li, C. C. Synergistic Manipulation of Zn^{2+} Ion Flux and Desolvation Effect Enabled by Anodic Growth of a 3D ZnF_2 Matrix for Long-Lifespan and Dendrite-Free Zn Metal Anodes. *Adv. Mater.* **2021**, *33*, 2007388.

10. Miao, Z.; Du, M.; Li, H.; Zhang, F.; Jiang, H.; Sang, Y.; Li, Q.; Liu, H.; Wang, S. Constructing Nano-Channeled Tin Layer on Metal Zinc for High-Performance Zinc-Ion Batteries Anode. *EcoMat* **2021**, *3*, e12125.

11. Ma, C.; Wang, X.; Lu, W.; Wang, C.; Yue, H.; Sun, G.; Zhang, D.; Du, F. Achieving Stable Zn Metal Anode via a Simple Nico Layered Double Hydroxides Artificial Coating for High Performance Aqueous Zn-Ion Batteries. *Chem. Eng. J.* **2022**, *429*, 132576.

12. Yang, Y.; Liu, C.; Lv, Z.; Yang, H.; Cheng, X.; Zhang, S.; Ye, M.; Zhang, Y.; Chen, L.; Zhao, J.; Li, C. C. Redistributing Zn-Ion Flux by Interlayer Ion Channels in MG-Al Layered Double Hydroxide-Based Artificial Solid Electrolyte Interface for Ultra-Stable and Dendrite-Free Zn Metal Anodes. *Energy Storage Mater.* **2021**, *41*, 230–239.

13. Guo, Z.; Fan, L.; Zhao, C.; Chen, A.; Liu, N.; Zhang, Y.; Zhang, N. A Dynamic and Self-Adapting Interface Coating for Stable Zn-Metal Anodes. *Adv. Mater.* **2021**, *34*, 2105133.

14. Cao, J.; Zhang, D.; Gu, C.; Wang, X.; Wang, S.; Zhang, X.; Qin, J.; Wu, Z. S. Manipulating Crystallographic Orientation of Zinc Deposition for Dendrite-Free Zinc Ion Batteries. *Adv. Energy Mater.* **2021**, *11*, 2101299.

15. Wu, C.; Xie, K.; Ren, K.; Yang, S.; Wang, Q. Dendrite-Free Zn Anodes Enabled by Functional Nitrogen-Doped Carbon Protective Layers for Aqueous Zinc-Ion Batteries. *Dalton Trans.* **2020**, *49*, 17629–17634.

16. Zou, P.; Zhang, R.; Yao, L.; Qin, J.; Kisslinger, K.; Zhuang, H.; Xin, H. L. Ultrahigh-Rate and Long-Life Zinc–Metal Anodes Enabled by Self-Accelerated Cation Migration. *Adv. Energy Mater.* **2021**, *11*, 2100982.

17. Cao, P.; Zhou, X.; Wei, A.; Meng, Q.; Ye, H.; Liu, W.; Tang, J.; Yang, J. Fast-Charging and Ultrahigh-Capacity Zinc Metal Anode for High-Performance Aqueous Zinc-Ion Batteries. *Adv. Funct. Mater.* **2021**, *31*, 2100398.

18. Hao, J.; Li, B.; Li, X.; Zeng, X.; Zhang, S.; Yang, F.; Liu, S.; Li, D.; Wu, C.; Guo, Z. An in-Depth Study of Zn Metal Surface Chemistry for Advanced Aqueous Zn-Ion Batteries. *Adv. Mater.* **2020**, *32*, 2003021.

19. Zhang, X.; Li, J.; Liu, D.; Liu, M.; Zhou, T.; Qi, K.; Shi, L.; Zhu, Y.; Qian, Y. Ultra-Long-Life and Highly Reversible Zn Metal Anodes Enabled by a desolvation and deanionization Interface Layer. *Energy Environ. Sci.* **2021**, *14*, 3120–3129.
